# Effectiveness of the Pfizer-BioNTech Vaccine against COVID-19-Associated Hospitalizations among Lebanese Adults ≥75 Years Old—Lebanon, April–May 2021

**DOI:** 10.3390/epidemiologia4020022

**Published:** 2023-06-14

**Authors:** Zeina Farah, Nadine Haddad, Hala Abou El Naja, Majd Saleh, Pamela Mrad, Nada Ghosn

**Affiliations:** 1 Epidemiological Surveillance Program, Ministry of Public Health, Beirut 2832, Lebanon; 2 Lebanon Country Office, World Health Organization, Beirut 2832, Lebanon

**Keywords:** COVID-19, BNT162 vaccine, hospitalization, case–control studies, Lebanon

## Abstract

In Lebanon, the nationwide vaccination against COVID-19 was launched in February 2021 using the Pfizer-BioNTech vaccine and prioritizing elderly people, persons with comorbidities, and healthcare workers. Our study aims to estimate the post-introduction vaccine effectiveness (VE) of the Pfizer-BioNTech vaccine in preventing COVID-19 hospitalizations among elderly people ≥75 years old in Lebanon. A case–control study design was used. Case patients were Lebanese, ≥75 years old, and hospitalized with positive PCR results during April–May 2021, and randomly selected from the database of the Epidemiological Surveillance Unit at the Ministry of Public Health (MOPH). Each case patient was matched by age and locality to two controls. The controls were hospitalized, non-COVID-19 patients, randomly selected from the MOPH hospital admission database. VE was calculated for fully (2 doses ≥14 days) and partially vaccinated (≥14 days of the first or within 14 days of the second dose) participants using multivariate logistic regression. A total of 345 case patients and 814 controls were recruited. Half were females, with a mean age of 83 years. A total of 14 case patients (5%) and 143 controls (22%) were fully vaccinated. A bivariate analysis showed a significant association with gender, month of confirmation/hospital admission, general health, chronic medical conditions, main income source, and living arrangement. After adjusting for a month of hospital admission and gender, the multivariate analysis yielded a VE of 82% (95% CI = 69–90%) against COVID-19-associated hospitalizations for those fully vaccinated and 53% (95% CI = 23–71%) for those partially vaccinated. Our study shows that the Pfizer-BioNTech vaccine is effective in reducing the risk for COVID-19-associated hospitalizations of Lebanese elderly people (≥75 years old). Additional studies are warranted to explore VE in reducing hospitalizations for younger age groups, as well as reducing COVID-19 infections.

## 1. Introduction

Since its emergence in December 2019, severe acute respiratory syndrome coronavirus 2 (SARS-CoV-2) has taken a tremendous toll on the population worldwide. By 4 May 2021, there have been over 153 million cases and 3.2 million deaths globally as a result of COVID-19 [1]. The implemented non-pharmacological interventions affected the daily lives of billions around the world, resulting in devastating socio-economic repercussions—the greatest challenge that humanity has ever faced since World War II [2].

In response to this pandemic, considerable efforts were made globally to develop effective and safe drugs and vaccines against SARS-CoV-2 [3]. Candidate vaccines were introduced with differing indications, contra-indications, and adverse events—each with a specific efficacy in preventing SARS-CoV-2 infection, severe outcomes, and death [4]. Hence, it is of paramount importance to evaluate the post-introduction effectiveness of these newly licensed and marketed vaccines, particularly among subgroups with underlying comorbidities, or those who may not have been included in pre-licensure vaccine trials, such as elderly people.

In Lebanon, a total of 529,205 COVID-19-confirmed cases and 7368 related deaths were reported as of 4 May 2021, since the detection of the first case on 21 February 2020 [5]. In January 2021, the Lebanese Ministry of Public Health (MOPH) issued its National Deployment and Vaccination Plan (NDVP) for COVID-19 vaccines, in which priority-target groups were selected based on specific risk factors, such as age, comorbidity, and occupation, in line with the World Health Organization (WHO) Strategic Advisory Group of Experts on Immunization (SAGE) recommendations [6]. On 14 February 2021, almost a year after the beginning of the outbreak, the national vaccination program was rolled out upon the arrival of the first batch of Pfizer-BioNTech vaccines targeting elderly healthcare workers and individuals with comorbidities [7]. As of 6 May 2021, a total of 498,722 vaccine doses of various types (Pfizer-BioNTech, AstraZeneca, Sputnik V, SinoPharm) were administered, as reported through the Inter-Ministerial and Municipal Platform for Assessment, Coordination and Tracking (IMPACT) platform [7]. The Pfizer-BioNTech vaccine was used for residents aged 75 years and older, with a time interval of 21 days between the 2 doses and a coverage of 37% for 2 doses, as of 6 May 2021.

As the nationwide vaccination progresses, it is vital to study the vaccine’s effectiveness in the community, as emphasized in the NDVP. Particularly, the national plan mentions the role of the Epidemiological Surveillance Unit (ESU) in studying the vaccine’s effectiveness in order to guide vaccination policies and public health and social measures [6].

Hence, our study aims to estimate the post-introduction effectiveness of the Pfizer-BioNTech COVID-19 vaccine against COVID-19 hospitalizations among Lebanese adults ≥75 years old, between 1 April and 31 May 2021.

## 2. Materials and Methods

### 2.1. Study Design

A case–control (CC) study design was conducted using structured questionnaires conducted via phone-call interviews. Our sample size was calculated using the precision method with 90% vaccine effectiveness for full vaccinations and 40% vaccine coverage, a ±5% precision value, and a type-1 error of 0.05. A minimum sample size of 318 case patients and 636 controls was needed [8]. The minimal sample size was multiplied by 50% to account for non-responses and refusals. Proportionate matching with 1:2 case patients to controls ratio was conducted according to the age groups ((75–85 years), (85–95 years)) and locality (governorate) of residence.

### 2.2. Study Population

The COVID-19 surveillance database of ESU-MOPH was screened to select confirmed patients reported as Lebanese, ≥75 years old, and hospitalized for COVID-19, confirmed by real-time polymerase chain reaction between 1 April and 31 May 2021. A random sample of 742 case patients was drawn using a random-number generator from the selected sampling frame and contacted to verify whether they met our study’s inclusion criteria. Case patients not meeting one of the inclusion criteria were discarded from our analysis.

The controls were randomly selected from the national database of hospital admissions covered by MOPH, which comprises around 50% of all hospitalizations in Lebanon [9]. The choice for this database was convenient since MOPH is usually the main funder for hospitalizations for the age group considered in our study. The selected controls were Lebanese patients, ≥75 years old, and hospitalized between 1 April and 31 May 2021 with admission diagnoses related to all International Classification of Diseases (ICD-10) chapters, excluding the COVID19 code. During our study period, any hospitalizations due to causes other than COVID-19 required a negative RT-PCR prior to the hospital admission; therefore, we assumed that the negative status of the controls was ascertained. The controls were later excluded if the investigation showed they did not meet one of our study’s inclusion criteria. The controls who were diagnosed with COVID-19 in the 3 months prior to their hospitalization were also excluded.

### 2.3. Variables

A structured questionnaire was used, including socio-demographic information (age, gender, place of residence, and main source of income), living conditions (number of household members, number of rooms, and living arrangements), health conditions in the 12-month period prior to their hospital admission (perception of general health status, presence of comorbidities, and ability to walk and climb), hospitalization status (duration of hospitalization, admission to the Intensive Care Unit (ICU), and duration of stay in the ICU, oxygen therapy, intubation, and discharge status), in addition to cognitive variables (ability to read and perform calculations). Furthermore, the crowding index was computed by dividing the number of household members by the number of rooms. The date of the PCR test-result confirmation was available for all case patients. However, the date of hospital admission was used for the controls as it was available in the MOPH hospital admission database. This time variable was considered a confounding factor as both the vaccination coverage and COVID-19 incidence showed time trends during the study period [5,7]. Three categories for hospital stay were generated: <3, 3 to 7, and over 7 days.

Participants self-reported their vaccination status by indicating their vaccination dates according to the SMS received from the MOPH vaccination platform. The vaccination data were only considered for participants reporting the exact dates of their vaccinations. Vaccination status included 4 categories: (1) “Unvaccinated”, defined as no receipt of the Pfizer-BioNTech vaccine before diagnosis/hospital admission; (2) “Single-dose vaccinated” <14 days before diagnosis/hospital admission; (3) “Partially vaccinated”, defined as receipt of 1 dose of the Pfizer-BioNTech vaccine ≥14 days before confirmation/hospital admission or 2 doses, with the 2nd dose received <14 days before confirmation/hospital admission; and (4) “Fully vaccinated”, defined as receipt of 2 doses of the vaccine with the 2nd dose received ≥14 days before confirmation/hospital admission.

### 2.4. Data Management

The collected data were digitalized using the DHIS2 tracker program and analyzed using R version 4.0.4 and R studio version 1.4.1103. For the VE analysis, fully and partially vaccinated participants were compared to unvaccinated subjects. Univariate descriptive statistics were used to assess the distribution of covariates among participants and identify the potential confounding factors. The characteristics of the case patients and controls were compared using chi-squared or Fisher’s exact tests for categorical variables, and Student’s *t*-test or Wilcoxon rank-sum tests for continuous variables. For the final selection of potential confounders to include in the logistic regression model, the “change-in-estimate” approach was used [8]. Covariates whose adjustments changed the crude odds ratio by ≥5% were included in the final models. The VE was estimated using the conditional logistic regression with the following formula [8]:

VE = (1 − matched, adjusted odds ratio (OR) for vaccination) × 100%

The 95% confidence intervals (CIs) of VE were calculated as 1 − CIOR, where CIOR is the confidence interval of the odds ratio estimates. 

### 2.5. Ethical Approval

The study was approved by the Institutional Review board of the Rafik Hariri University Hospital. Informed consent was obtained from the study subjects prior to their participation.

## 3. Results

### 3.1. Study Population

**Figure 1:**
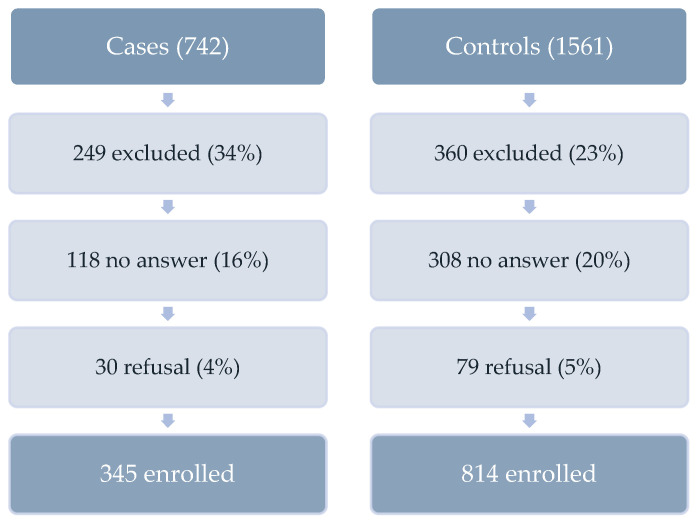
Selection of study participants and sample sizes, VE CC study, ≥75 years old; April–May 2021, Lebanon.

Between 1 April and 31 May 2021, 742 case patients and 1561 controls were contacted for the study. However, due to either being excluded for not fitting the case/control definition (34%; 23%), no reply (16%; 20%), or a refusal to participate (4%; 5%), the obtained number of participants was 345 case patients to 814 matched controls (Figure 1). The mean ages of the participating case patients and controls were 83.1 ± 5.6 and 82.8 ± 5.7 years, respectively. The highest proportion of case patients and controls resided in the Mount Lebanon governorate (37%; 39%). Case patients had an equal proportion of females and males (50%); however, the control group had a significantly higher proportion of females (57%) (*p* = 0.03) (Table 1). Concerning the household arrangements, case patients had more rooms (3.4 ± 1.4) compared to the controls (3.0 ± 1.2) and a higher number of household members (2.4 ± 1.7) compared to the controls (2.0 ± 1.9) (*p* < 0.001). Most case patients and controls (87%; 76%) lived with family members. No significant differences were noted for the crowding index between our comparison groups (*p* = 0.64) (Table 1). For the vast majority in both groups, family support was the main source of income. However, case patients (11%) had more retirement pensions than the controls (2%) (Table 1). Case patients (54%, *n* = 184) were significantly more likely than the controls (32%, *n* = 233) to perform calculations without difficulty. They were also more likely to read and write without difficulty (49% of case patients as compared to 33% among the controls, *p* < 0.001). On the other hand, the proportion of subjects reporting an inability to read or write was higher among the controls (43%, *n* = 329) than the case patients (27%, *n* = 92) (Table 1). The general health status of the case patients and controls in the 12 months prior to their hospital admission was also significantly different (*p* < 0.001) and was mostly good (44% for case patients and 36% for controls), followed by a fair health status (26% for case patients and 32% for controls). A total of 41% of case patients reported no difficulty in walking or climbing up or down the stairs prior to their hospital admission, while 34% of controls did not have the ability to conduct this task (Table 1). The majority of the case patients and controls had at least one underlying condition (85%; 92%) (*p* = 0.001). The underlying conditions reported for both the case patients and controls were mostly hypertension (70%; 76%) followed by heart diseases (50%; 66%) and diabetes (43%; 47%), respectively (Table 1).

### 3.2. Hospitalization

As for the hospitalization information, the mean duration of hospital stays was significantly longer among case patients (11.1 ± 9.3 days) compared to the controls (6.0 ± 7.0 days). In particular, the length of stay was mostly >7 days for case patients (53%), while it was 3–7 days for the controls (60%). The majority of case patients required ICU admissions (67%) with a longer duration of stay (8.5 ± 7.9 days), while 25% of the controls were admitted to the ICU (*p* < 0.001) (Table 2). Furthermore, case patients were significantly more likely than the controls to require oxygen therapy (90%) and intubation (46%) (*p* < 0.001). Death upon discharge was significantly higher among case patients (52%), compared to the controls (10%) (*p* < 0.001). Among the case patients, the majority of deaths were due to COVID-19 (93%) (Table 2). Of note, no change in the abovementioned results was observed when restricting the univariate analysis to participants with complete vaccination data.

### 3.3. Vaccination Effectiveness

The majority of the case patients and controls reported having no vaccination (81%; 63%), 13% of case patients reported having received one dose compared to 11% for the controls, and 5% of case patients reported having two doses compared to 27% of the controls. The majority of case patients and controls reported no side effects following their first (66%; 82%) and second (67%; 81%) doses (*p* = 0.002 and 0.13, respectively) (Table 3).

Taking fully vaccinated individuals 14 days after their second dose, 14 case patients (4%) and 143 controls (21%) were fully vaccinated and the crude OR was 0.16 (95% CI = 0.09–0.28). After adjusting for the month of hospital admission and gender, the multivariate analysis yielded an adjusted OR of 0.18 and a VE of 82% (95% CI = 69–90%) (Table 3). For those partially vaccinated, the crude OR was 0.49 (95% CI = 0.30–0.79). After adjusting for the month of hospital admission, the adjusted OR was 0.47 and the vaccine’s effectiveness was 53% (95% CI = 23%–71%) (Table 4).

On the other hand, there was no significant effect for receiving the first dose of the vaccine within 14 days of confirmation/admission (adjusted OR = 1.09 and 95% CI = 0.63–1.87).

## 4. Discussion

Clinical trials assessed the efficacy of the Pfizer-BioNTech vaccine against COVID-19-associated infections; however, monitoring the efficacy against COVID-19-associated hospitalizations in the clinical trials was challenging because few COVID-19 patients needed hospitalization [10]. Our study supported the assessment of COVID-19 hospitalizations among a high-risk group. In our analysis of Lebanese ≥75 years old, hospitalized between 1 April and 31 May 2021, vaccination with Pfizer-BioNTech was significantly less likely among hospitalized patients with COVID-19 than other conditions. These results are consistent with the available evidence showing a reduction in COVID-19-associated hospitalizations among vaccinated compared to unvaccinated patients [11]. This highlights the importance of vaccinations against COVID-19 in reducing severe outcomes in this high-risk group.

In this study, the multivariate analysis of hospitalized patients ≥75 years old revealed that the Pfizer-BioNTech vaccine was associated with significant protection against COVID-19 hospitalizations.

Our results suggest that two doses of the Pfizer-BioNTech vaccine, at least 14 days after the vaccine administration, provided a substantial level of protection (82% VE) against hospitalization for elderly individuals (≥75years) in Lebanon between April and May 2021. These results are consistent with previous studies, mainly two studies conducted in the United States (US) during March–July and February–August 2021 targeting adults, showing the VE of mRNA vaccines (Pfizer-BioNTech and Moderna) against hospitalizations to be 86% (95% CI = 82–88%) and 80% (95% CI = 68–87%) among fully vaccinated individuals aged 65 years and older [12,13]. It is also comparable to the results of a study conducted in the US showing the VE of mRNA vaccines against hospitalizations among fully vaccinated participants aged 18 years and older to be 85% (95% CI = 82% to 88%) during the Alpha-variant period [14].

Moreover, in assessing the impact of one dose of the vaccine, no significant effectiveness within 14 days of the first dose was detected. This is also in line with results from other studies showing no significant effect in the 14 days after the first dose [10,15].

However, our results are lower than the VE reported in some studies assessing the effectiveness of mRNA vaccines against COVID-19 hospitalizations, mainly in two studies conducted during January–March and March–August 2021. These studies targeted adults in the US showing the VE of mRNA vaccines (Pfizer-BioNTech and Moderna vaccines) for full vaccinations to be 94% (95% CI = 49–99%) for adults ≥65 years old and the VE of the Pfizer-BioNTech vaccine to be 91% (95% CI = 88–93%) among adults ≥18 years old, respectively [10,16]. Similarly, our results are lower than the findings of a study conducted in Canada showing a VE of 91% (95% CI = 87–94%) against Alpha-variant hospitalizations among those 60 years old and older, 7 days after receiving the second dose of the Pfizer-BioNTech vaccine [17].

As for the effectiveness of partial vaccinations, our results show the effectiveness of 53% among those who were partially vaccinated (having received 1 dose of the vaccine ≥14 days before confirmation/hospital admission, or 2 doses with the second dose being received <14 days after confirmation/hospital admission). These results are lower than those reported in a study conducted in the US showing the Pfizer-BioNTech vaccine’s VE to be 64% (95% CI = 28–82%) against hospitalization (after 14 days of the first dose or within 14 days of the second dose) among adults ≥65 years old [10]. It was also lower than those reported in other studies assessing the VE against hospitalization after 14 days of one Pfizer-BioNTech dose, showing a VE of 71% (95% CI = 47–91%) among elderly people ≥80 years old in the UK and a VE of 70% (95% CI = 60–77%) among people ≥16 years old in Canada [18,19].

The difference between the study results may be mostly due to the mean age group used in this study, which is around 83 years old, which is older in comparison to the other studies. Moreover, the fact that some of these studies assessed a combination of mRNA vaccines meant that not only the Pfizer-BioNTech vaccine could have affected the comparison. Other factors to mention include the differences in the study design, evaluated population, and inclusion criteria between the studies, as well as the variability in unmeasured confounding factors.

Moreover, our study occurred between April and May 2021 where the dominant variant in Lebanon was suggested to have been the Alpha variant. In Lebanon, genomic sequencing began in June, with the Alpha variant being the dominant circulating virus [20]; the SARS-CoV2 Delta variant became the dominant circulating virus in Lebanon in July 2021. Therefore, it is crucial to interpret the VE results and cautiously compare them with other international studies, taking into account SARS-CoV2 circulating variants at the time of study.

Furthermore, in our study design, targeting elderly individuals older than 75 years of age for both the case patients and controls might have affected vaccine uptake values as well as elderly individuals’ exposure to infection in the sense that their decreased mobility might have affected their accessibility to vaccines; likewise, elderly individuals, due to their vulnerability, might have limited social interactions, hence decreasing the risk of exposure to the infection during the pandemic. Any of these factors might have biased our VE estimates.

Additionally, the majority of case patients and controls reported having no vaccination dose (90%; 64%) at the time of study, which can be explained by the timeline of the vaccine rollout in the country that started in mid-February and that initially faced delays due to the interruptions in receiving vaccine batches into the country.

On the other hand, the important results to be highlighted are the severity of illness of COVID-19-associated hospitalizations compared to non-COVID-19-associated hospitalizations. In our study, although case patients subjectively reported having a better score for their general health status compared to the controls, their prognosis was more severe than the controls, with longer durations of stay, a significantly greater need for intensive care, oxygen therapy and intubation, and ultimately death.

As vaccinations may provide a false sense of security, our results highlight the importance of adhering to public health measures to avoid COVID-19-associated hospitalizations where vaccinated individuals are still advised to continue practicing hand hygiene, physical distancing, and mask wearing [21].

The study’s results are subject to some limitations. First, the MOPH hospital’s admission database, from where the controls were sampled, was not inclusive of the total target population as it covers the hospitalization of citizens who are uninsured and who usually belong to the most deprived groups of the population, such as seasonal workers, farmers, and retired and unemployed persons; thus, on average, an older and poorer population [9]. This was reflected by some indicators measured in the study, such as household arrangements, source of income, and mobility and cognitive statuses, which showed a difference between the controls and cases. Additionally, vaccine effectiveness estimates might be confounded by certain unmeasured behaviors, such as adherence to non-pharmaceutical interventions, including mask use or the recent attendance of gatherings, in addition to other variables, such as socio-economic status and prior SARS-CoV-2 infection. This might have affected our results as uncontrolled confounders might lead to differences in vaccine uptake numbers, exposure to infection, and the development of severe disease implications.

## 5. Conclusions

Our study showed that the Pfizer-BioNTech vaccine is effective in reducing the risk for COVID-19-associated hospitalizations in older adults. These results reinforce the importance of vaccinations among the elderly who are at high risk of COVID-19 hospitalizations. They can be used to promote COVID-19 vaccinations and reduce individuals’ hesitancy to receive the vaccine. Additional studies are warranted to explore the effectiveness of the vaccine to reduce hospitalizations of younger age groups, as well as reducing COVID-19 infections by taking into account other vaccine products in light of the emergence of new SARS-CoV-2 variants and the increase in the elapsed time since administering the vaccinations.

## Figures and Tables

**Table 1:** Characteristics of Lebanese, hospitalized, COVID-19 case patients and controls aged ≥75 years; April–May 2021.

Characteristics			Case Patients (N = 345) * n (%)	Controls (N = 814) * n (%)	*p*-Value
Socio-demographic characteristics					
Age (mean ± sd)			83.1 ± 5.6	82.8 ± 5.7	0.53
Age groups	75–84		236 (68.4)	559 (68.7)	0.98
	85+		109 (31.6)	255 (31.3)	
Gender	Female		171 (49.6)	463 (56.9)	0.03
	Male		174 (50.4)	350 (43.1)	
Place of residence (governorate)	Mount Lebanon		128 (37.1)	318 (39.0)	0.33
	Bekaa/Baalbeck-Hermel		75 (21.7)	138 (17.0)	
	South Nabatieh		64 (18.6)	178 (21.9)	
	North		47 (13.6)	110 (13.5)	
	Beirut		31 (9.0)	70 (8.6)	
Main income source	Family help		265 (77.5)	646 (82.8)	<0.001
	Retirement pension		38 (11.1)	19 (2.4)	
	Personal savings		34 (9.9)	77 (9.9)	
	Financial help (not family)		4 (1.2)	30 (3.9)	
	No income		1 (0.3)	8 (1.0)	
Living conditions					
Number of household members (mean ± sd)			2.4 ± 1.7	2.0 ± 1.9	<0.001
Number of rooms (mean ± sd)			3.4 ± 1.4	3.0 ± 1.2	<0.001
Crowding index (mean ± sd)			0.8 ± 0.6	0.7 ± 0.7	0.64
Living arrangement	Alone		24 (7.0)	122 (15.1)	<0.001
	With Family		299 (87.1)	610 (75.5)	
	With domestic help		17 (5.0)	61 (7.5)	
	Long-term facility		3 (0.9)	15 (1.9)	
Health conditions (in the 12 month period prior to hospital admission)					
Perception of general health status	Very good		61 (17.9)	51 (6.4)	<0.001
	Good		151 (44.4)	290 (36.1)	
	Fair		87 (25.6)	258 (32.1)	
	Poor		34 (10)	169 (21.0)	
	Very poor		7 (2.1)	35 (4.4)	
Ability to walk, climb up or down stairs alone	Yes, without difficulty		141 (41.2)	162 (20.4)	
	Yes, but with some difficulty		90 (26.3)	161 (20.2)	<0.001
	Yes, but with help or assistance		73 (21.4)	202 (25.4)	
	No		38 (11.1)	271 (34.0)	
Underlying conditions	Hypertension		221 (69.9)	549 (75.6)	0.07
	Heart disease		150 (50.2)	448 (66.1)	<0.001
	Diabetes		125 (42.7)	314 (47.0)	0.24
	Kidney disease		34 (12.6)	76 (13.0)	0.96
	Lung disease		30 (10.9)	111 (18.3)	0.007
	Cancer		12 (4.4)	68 (11.3)	0.002
	Asthma		10 (3.7)	30 (5.2)	0.46
	Rheumatological disorders		6 (2.2)	45 (7.8)	0.003
	Liver disease		4 (1.5)	15 (2.6)	0.44
	History of cancer		4 (1.5)	25 (4.3)	0.05
	Immunodeficiency		4 (1.5)	6 (1.0)	0.82
Presence of at least one underlying condition	No		50 (15.1)	65 (8.4)	0.001
	Yes		282 (84.9)	713 (91.6)	
Cognitive status (in the 12 month period prior to hospital admission)					
Ability to read and write	Yes, without difficulty		169 (49.4)	250 (32.5)	<0.001
	Yes, but with some difficulty		53 (15.5)	142 (18.5)	
	Yes, but with help or assistance		28 (8.2)	48 (6.2)	
	No		92 (26.9)	329 (42.8)	
Ability to perform calculations	Yes, without difficulty		184 (53.8)	233 (31.6)	<0.001
	Yes, but with some difficulty		50 (14.6)	91 (12.3)	
	Yes, but with help or assistance		21 (6.1)	53 (7.2)	
	No		87 (25.5)	361 (48.9)	

* The following variables have missing observations: main income source (3 cases and 34 controls), living arrangement (3 cases and 6 controls), perception of general health status (5 cases and 11 controls), ability to walk or climb up or down stairs alone (3 cases and 18 controls), presence of at least one underlying condition (13 cases and 36 controls), ability to read and write (3 cases and 45 controls), ability to perform calculations (3 cases and 76 controls).

**Table 2:** Hospitalization data for Lebanese, hospitalized, COVID-19 case patients and controls aged ≥75 years; April–May 2021.

Characteristics		Case Patients (N = 345) ^§^ *n* (%)	Controls (N = 814) ^§^ *n* (%)	*p*-Value
Month of confirmation/hospital admission *	April	271 (78.6)	464 (57.0)	<0.001
	May	74 (21.4)	350 (43.0)	
Duration of hospitalization (days)		11.1 ± 9.3	6.0 ± 7.0	<0.001
Hospital stay	<3 days	31 (9.6)	160 (22.3)	<0.001
	3–7 days	120 (37.0)	426 (59.5)	
	>7 days	173 (53.4)	130 (18.2)	
Admission to ICU	Yes	224 (66.9)	180 (24.6)	<0.001
	No	111 (33.1)	551 (75.4)	
Duration of stay in ICU		8.5 ± 7.9	5.8 ± 5.8	<0.001
Oxygenotherapy	Yes	275 (90.2)	175 (25.8)	<0.001
	No	30 (9.8)	503 (74.2)	
Mechanical ventilation	Yes	107 (46.3)	40 (6.1)	<0.001
	No	124 (53.7)	611 (93.9)	
Discharge status	Alive	153 (48.3)	711 (89.8)	<0.001
	Death	164 (51.7)	81 (10.2)	
Cause of death **	COVID-19	136 (93.4)	0 (0)	<0.001
	Other causes	9 (6.2)	70 (100)	

* Month of confirmation for case patients and hospital admissions for controls. ^§^ Some variables have missing observations: duration of hospitalization (21 cases and 98 controls), admission to ICU (10 cases and 83 controls), oxygenotherapy (40 cases and 136 controls), mechanical ventilation (114 cases and 163 controls), discharge status (28 cases and 22 controls), cause of death (19 cases and 11 controls). ** Among deaths.

**Table 3:** Vaccination data for Lebanese, hospitalized, COVID-19 case patients and controls aged ≥75 years; April–May 2021 *.

Characteristics		Case Patients (N = 337) *n* (%)	Controls (N = 695) *n* (%)	*p*-Value
Number of received doses prior to hospital admission	Two doses	18 (5.3)	184 (26.5)	<0.001
	One dose	45 (13.4)	73 (10.5)	
	Zero doses	274 (81.3)	438 (63.0)	
Vaccination status prior to confirmation/hospital admission	Fully vaccinated	14 (4.2)	143 (20.6)	<0.001
	Partially vaccinated	24 (7.1)	79 (11.4)	
	Within 14 days of first dose	25 (7.4)	35 (5.0)	
	Unvaccinated	274 (81.3)	438 (63.0)	
Adverse events following first dose	No side effects	41 (66.1)	210 (81.7)	0.002
	Minor side effects	10 (16.1)	34 (13.2)	
	Moderate side effects	5 (8.1)	10 (3.9)	
	Severe	6 (9.7)	3 (1.2)	
Adverse events following second dose	No side effects	12 (66.7)	145 (80.6)	0.13
	Minor side effects	3 (16.7)	26 (14.4)	
	Moderate side effects	2 (11.1)	5 (2.8)	
	Severe	1 (5.5)	4 (2.2)	

* Participants with no vaccination dates were excluded: 8 case patients and 119 controls.

**Table 4:** The Pfizer-BioNTech vaccine’s effectiveness * against COVID-19 among Lebanese, hospitalized, COVID-19 case patients and controls aged ≥75 years; April–May 2021 (*n* = 1032).

Vaccination Status	Case Patients	Controls	Unadjusted OR (95% CI)	VE against COVID-19 Hospitalizations (95% CI)
Fully vaccinated	14 (4.2)	143 (20.6)	0.16 (0.09–0.28)	82 (69–90) *
Partially vaccinated	24 (7.1)	79 (11.4)	0.49 (0.30–0.79)	53 (23–71) **

* Adjusted for month of confirmation/hospital admission and gender. ** Adjusted for month of confirmation/hospital admission.

## Data Availability

The data presented in this study are available on request from the corresponding author. The data are not publicly available due to restrictions related to public institution policies.
